# Robust All-Solid-State Batteries with Sodium Ion Electrolyte, Aluminum and Additive Manufacturing Inconel 625 Electrodes

**DOI:** 10.3390/molecules30224465

**Published:** 2025-11-19

**Authors:** Manuela C. Baptista, Antonio B. Vale, Jose M. Costa, Maria Helena Braga

**Affiliations:** 1 Faculty of Engineering, University of Porto, Rua Dr. Roberto Frias, 4200-465 Porto, Portugal; up200501276@up.pt (M.C.B.); up201709900@up.pt (A.B.V.); jose.costa@fe.up.pt (J.M.C.); 2 MatER—Materials for Energy Research Laboratory, Faculty of Engineering, University of Porto, Rua Dr. Roberto Frias, 4200-465 Porto, Portugal; 3 LAETA, Institute of Science and Innovation in Mechanical and Industrial Engineering, Rua Dr. Roberto Frias, 4200-465 Porto, Portugal

**Keywords:** all solid-state batteries, Na ion batteries, electrode less batteries, Inconel 625, ferroelectric electrolyte

## Abstract

This study investigates all-solid-state batteries employing multifunctional metallic current collectors/electrodes that remain electrochemically inert toward an alkali-based Na ion solid electrolyte. Inconel 625 was evaluated as the positive current collector in combination with aluminum as the negative electrode and the ferroelectric electrolyte Na2.99Ba0.005OCl. The inertness of both electrodes enabled the construction of a robust device architecture that behaved as a true battery, exhibiting a two-phase equilibrium discharge plateau at ~1.1 V despite the absence of traditional Faradaic reactions. After a one-month rest period, the cell was sequentially discharged through external resistors and retained full functionality for one year. Cyclic voltammetry confirmed a stable electrochemical response over repeated cycling. The final long-term discharge under a 9.47 kΩ load produced a steady ~0.92 V plateau and delivered a total capacity of 35 mAh (~2.3 mAh·cm^−2^). Post-mortem analyses revealed excellent chemical and mechanical stability of Inconel 625 after extended operation, while aluminum showed superficial surface degradation attributed to residual moisture, with X-ray diffraction indicating the formation of aluminum hydroxide. Scanning Kelvin probe measurements guided electrode selection and provided insight into interfacial energetics, whereas scanning electron microscopy confirmed interface integrity. Complementary density functional theory simulations optimized the crystalline bulk and surfaces of Inconel, demonstrating interfacial stability at the atomic scale. Overall, this work elucidates the fundamental driving forces underlying traditional battery operation by studying a “capacity-less” system, highlighting the central role of interfacial electrostatics in sustaining battery-like discharge behavior in the absence of redox-active electrodes.

## 1. Introduction

The development of new materials and technologies must be guided by a critical assessment of their environmental impact and long-term sustainability [1,2]. In the current era of energy transition, driven by the urgent need to mitigate climate change, solutions such as zero-emission transportation have been widely promoted [3,4]. However, the true effectiveness of these solutions should be evaluated not only in terms of operation but also in how materials are selected, processed, and integrated throughout the device’s lifetime [5,6].

In the batteries field in particular (Table 1), careful material selection is essential to ensure performance, longevity, and safety [7,8,9,10,11,12,13]. One configuration that has gained substantial traction in recent years is the anode-less architecture, in which the anode is formed in situ during operation, a concept that has intensified interest in the field and prompted numerous detailed analyses and review contributions [14,15,16,17,18,19,20,21,22,23] (Table 1). Compatibility between components, especially when they are in direct contact, is a critical design factor: undesired chemical or structural interactions can trigger premature degradation and thereby compromise system efficiency and stability [24]. The advancement of sustainable battery technologies therefore depends on rational materials engineering strategies that prioritize long-term chemical, structural, and interfacial stability to enable reliable and environmentally responsible energy solutions [25,26].

The manufacturing approach used for component fabrication and device assembly also plays a pivotal role in determining the overall environmental and operational efficiency of a system. Key factors—such as minimizing material waste, reducing energy and water consumption, and decreasing human labor intensity—must be considered to achieve efficient manufacturing [27,28]. Metal Additive Manufacturing (AM) has transformed the production of complex components by enabling layer-by-layer fabrication directly from digital models, thereby overcoming the geometric and material limitations associated with traditional subtractive techniques [29]. This paradigm shift introduces significant advantages, including increased design freedom, reduced waste, and the ability to fabricate intricate geometries that are difficult or impossible to produce by conventional methods [30,31,32,33,34].

While powder-based AM technologies, such as powder bed fusion and directed energy deposition, are well established, ongoing innovation has expanded the AM landscape with more accessible and cost-efficient alternatives. Among these, metal Fused Filament Fabrication (FFF) has emerged as a promising extrusion-based technique [35,36]. Unlike powder-bed systems, metal FFF uses composite filaments consisting of metal particles embedded in a polymer binder, deposited layer by layer through a process similar to polymer FFF. After printing, components undergo debinding to remove the polymer and sintering to densify the metallic structure [37,38]. This approach provides a safer, lower-cost alternative to powder-bed fusion while maintaining the capability to produce fully functional metal parts with complex geometries [38].

To fully exploit the capabilities of such AM techniques, particularly metal FFF, Design for Additive Manufacturing (DfAM) is essential. DfAM encompasses design principles tailored specifically to additive processes, extending beyond the constraints of conventional manufacturing [37,39,40]. It includes strategies such as topology optimization, lattice and cellular architectures, support minimization, and functionally graded materials [41]. In metal AM, these approaches allow precise control over material placement, enabling components that are simultaneously lightweight and mechanically robust [42]. DfAM also contributes to mitigating thermal stresses and geometric distortions intrinsic to metal AM, reducing the need for extensive post-processing. These capabilities are of particular relevance in sectors where performance, efficiency, and weight reduction are critical, such as aerospace, automotive, and energy storage [40].

The combined application of metal AM and DfAM in energy harvesting and storage devices presents significant opportunities for improving both performance and longevity [43]. One of the central challenges in the design of solid-state batteries is achieving effective interfaces between electrodes and solid electrolytes [44]. Increasing the contact area between these components is essential for facilitating efficient ion transfer and enhancing electrochemical performance [45]. Conventional manufacturing methods impose geometric limitations that hinder interface optimization, whereas DfAM enables the creation of complex, high-surface-area features that enhance electrode–electrolyte interaction and, consequently, device performance [46,47].

Among the metallic materials explored for FFF, high-performance alloys have gained particular attention for demanding applications. Inconel 625, a nickel-based superalloy, is notable for its exceptional resistance to oxidation and corrosion in high-temperature and aggressive environments [35,48,49,50,51]. Widely employed in aerospace, marine, and energy sectors, Inconel 625 combines superior mechanical strength with excellent thermochemical stability. It is especially resistant to corrosion in contact with alkali electrolytes, making it a suitable current collector (CC) material for energy storage devices [52,53]. Moreover, its resistance to creep and thermal fatigue under cyclic thermal loads further enhances its durability in such systems [54].

Solid electrolytes exhibiting ferroelectric behaviour, such as Na_2.99_Ba_0.005_OCl, represent a unique class of multifunctional materials capable of simultaneously enabling energy harvesting and storage. This compound, belonging to the Na_3_OCl family, incorporates low-concentration substitution of two Na^+^ ions by one Ba^2+^ ion, inducing local lattice distortions that enhance ferroelectricity [55]. In a symmetric Au/Na_2.99_Ba_0.005_OCl/Au cell, the electrolyte exhibits an ionic conductivity of ~20 mS.cm^−1^ and an exceptionally high dielectric constant $\varepsilon_{r}$ ≈ 8 × 10^7^; in asymmetric configuration, it reaches a maximum capacitance of $C_{max}$ ≈ 15 F.cm^−2^ at 25 °C [56]. These features allow its use not only as an efficient ionic conductor but also as an active functional material capable of piezoelectric and pyroelectric response, leading to electromechanical coupling [55,57].

In this work, an integrated energy harvesting and storage device was developed incorporating the ferroelectric electrolyte Na_2.99_Ba_0.005_OCl, demonstrating its potential as a key multifunctional component for enhanced device performance. To investigate the morphological, compositional, and electrochemical characteristics of the materials throughout different processing stages, complementary characterization techniques were employed. Scanning Kelvin Probe (SKP) was used to map surface potential; Scanning Electron Microscopy with Energy-Dispersive X-ray spectroscopy (SEM/EDX) provided insights into surface morphology and elemental distribution; and X-ray diffraction (XRD) indicated the possible formation of an aluminum hydroxide phase, elucidating secondary phase evolution during processing. Electrochemical behavior was assessed by Potentiostatic Electrochemical Impedance Spectroscopy (PEIS), Cyclic Voltammetry (CV), and charge/discharge measurements, enabling detailed analysis of charge-transport mechanisms and energy-storage performance. In parallel, Density Functional Theory (DFT), implemented in VASP, was used to optimize the crystalline bulk and surfaces of Inconel with different stoichiometries, providing atomic-scale insights into surface energetics and their impact on electrolyte–current-collector interactions. Additionally, Inconel 625 was fabricated using metal FFF, integrating DfAM principles to enhance interfacial contact between the CC and the solid electrolyte.

Finally, this study aims to elucidate the role of interfaces in energy storage through a proof-by-contradiction (reductio ad absurdum) approach. By employing unconventional electrodes inert to Na^+^ from the electrolyte, it is possible to fabricate a cell with no initial capacity that nonetheless exhibits a persistent flat discharge plateau lasting for months. This counterintuitive behavior highlights the fundamental role of interfacial phenomena in sustaining charge storage and transport.

**Table 1:** Comparative study with related representative technologies battery cells in the literature.

Cell Configuration	Electrolyte	Open-Circuit Voltage/Voltage Drift Behavior	Cycle Stability/Duration	Ref.
Anode-less half-cell: Li//Cu	Nb-LLZO	**Initial OCV:** ~0 V **Voltage Drift:** The initial Li nucleation shows a sharp voltage drop to -65 mV, followed by a stable growth plateau at around -45 mV, with a gradual polarization increase during cycling due to void formation and dead Li.	**Stability:** The cells demonstrated cycling stability over hundreds of hours (≈180 h until short circuit for the 50 nm Cu configuration) at current densities of 0.05–0.1 mA.cm^−2^. Coulombic efficiency stabilized in the range of 75–90% in later cycles. **Capacity:** A fixed areal capacity of 0.1 mAh.cm^−2^ was used for stripping/plating cycles. The performance and failure mechanisms were highly dependent on the current collector thickness, influencing the achievable cycle life before short-circuiting.	[58]
Anode-less half-cell: Li//stainless steel current collector	LPSCl	**Initial OCV:** ~0 V **Voltage Drift:** A sharp voltage drops to ~0.25 V occurs during initial lithium nucleation, followed by a stable plating plateau at ~0.05 V. Increasing stack pressure from 2 to 10 MPa reduces initial polarization and extends the stable voltage plateau, enabling higher plating capacities before failure.	**Stability:** The failure mechanism shifts with stack pressure: at low pressure (2–5 MPa), failure is caused by irregular Li plating; at high pressure (20 MPa), failure is dominated by mechanical fracture induced at surface notches of the solid electrolyte. **Capacity:** The maximum areal capacity before short circuit is highly dependent on stack pressure, increasing from 1.24 mAh.cm^−2^ at 2 MPa to a maximum of 4.56 mAh.cm^−2^ at 10 MPa, before dropping significantly to 2.44 mAh·cm^−2^ at 20 MPa due to pressure induced fracture.	[59]
Full cell Zn//ZnI_2_	DCHE—Dual-Confinement Hydrogel Electrolyte	**Initial OCV:** ~1.3 V **Voltage Drift:** Minimal voltage drift with 93.5% capacity retention after 48 h open-circuit stand.	**Stability:** The cell demonstrated exceptional long-term stability over 6000 h (2500 cycles), maintaining high operational integrity throughout the test. **Capacity:** The cell achieved an outstanding capacity retention of 88.9% when cycled at a current density of 100 mA.g^−1^.	[60]
Full cell Na//ZnCl_2_	Dual-phase (β″—Al_2_O_3_ solid electrolyte + NaAlCl_4_ molten salt catholyte)	**Initial OCV:** ~1.92 V–2.13 V (dependent on operating temperature). **Voltage Drift:** Stable charge/discharge plateaus observed over 56 cycles at 280 °C with minimal polarization growth. Performance degradation and increased polarization were significantly more rapid at 240 °C due to the absence of beneficial liquid-phase formation.	**Stability:** Stable performance attributed to liquid-phase formation (NaCl-ZnCl_2_) that suppresses Zn and NaCl particle growth. Cells operated at 240 °C showed significantly faster degradation due to solid-state reactions only. **Capacity:** ~65 mAh (cycling between 48 and 90% SOC) and ~110 mAh (deep cycling between 20 and 90% SOC) at 280 °C. active cell area: 3 cm^2^	[61]
Electrodeless Cell (Al//Inconel 625)	Na_2.99_Ba_0.005_ClO The capacity of the cell is solely dependent on the sodium concentration on the electrolyte	**Initial OCV:** ~1.06 V (after assembly). **Voltage Drift:** Stable discharge plateau at ~1.1 V observed for months under different resistances; after one year, OCV decayed to ~0.93 V. Self-charging events observed during discharge (e.g., voltage increased from 0.79 V to 0.94 V under 9.74 kΩ load).	**Stability:** Cell remained functional for over one year under continuous and sequential discharge through various external resistors, demonstrating exceptional long-term stability. Inconel 625 showed no significant degradation; aluminum exhibited superficial mechanical degradation due to residual moisture. **Capacity:** Discharge capacity of 35 mAh (~2.3 mAh.cm^−2^) under 9.47 kΩ load.	This work

## 2. Results and Discussion

The system under study is based on a ferroelectric solid electrolyte, Na_2.99_Ba_0.005_OCl, whose spontaneous polarization and charge storage capability arise from the oriented arrangement of ions within the material. The combination of this electrolyte with two dissimilar metals, aluminum (negative acting electrode) and Inconel 625 (positive acting electrode) introduces an electrochemical potential $\bar{\mu }$ difference corresponding to the difference in chemical potentials, μ. To spontaneously equilibrate the difference in electrochemical potentials of the materials in electrical contact ${\bar{\mu }}_{electrode}-{\bar{\mu }}_{electrolyte}=0=\mu_{electrode}-\mu_{electrolyte}+z_{{Na}^{+}}e(\phi_{electrode}-\phi_{electrolyte})$, where $z_{{Na}^{+}}=1$ is the valency of the mobile ion, e is the unitary charge, and ϕ the surface potential, the formation of electrical double layer capacitors (EDLC) takes place [62]. As the electrolyte is an insulator and the Al/Na_2.99_Ba_0.005_OCl/Inconel 625 cell is electrode less—it may not exchange ions or electrons—the only way to equilibrate the electrochemical potentials is by forming an EDLC, $\mu_{electrode}-\mu_{electrolyte}=-e(\phi_{electrode}-\phi_{electrolyte})$, where $\phi_{electrode}-\phi_{electrolyte}=\Delta V$ [62]. The potential difference in the cell is the sum of the ΔV at the interfaces’ electrode/electrolyte. The plateau is formed by sodium plating from the electrons on the surface of the electrodes and ions in the electrolyte ${Na}^{+}+\bar{e}↔Na(s)$ [56,57]. The Na(s) proceeds from the electrolyte and is protected and eventually oxidized by it [57].

As the electrolyte is a ferroelectric, a nonlinear system with potential for self-oscillatory behavior develops, supported by: ***(i)*** a spontaneous internal field generated by ferroelectric polarization; ***(ii)*** Na^+^ migration via interstitial hopping or vacancy-mediated mechanisms; and ***(iii)*** asymmetric metal/electrolyte interactions.

This section provides a comprehensive characterization of both the individual components and the assembled electrochemical device. The analysis begins with SKP measurements of the Inconel 625 surface, followed by electrochemical characterization of the complete device under operating conditions. *Post-mortem* SKP analyses were then conducted on the Inconel after disassembly to evaluate possible surface modifications. Finally, SEM/EDX analyses were performed to investigate the morphology and elemental composition of the CC.

With an electrode-less battery where no reactions take place with the positive electrode/current collector while discharging, understanding the role of the collectors as chemical potential holders, and origin of a stable 48 h, or even ~800 h, flat plateau at ~1 V with an output current that may reach ~0.1 mA (~0.1/15 mA.cm^−2^) is paramount. After 11 months cycling with different resistors, the cell charges for 15 min to 2.1 V and subsequently uninterrupted discharges with a ~10 kΩ for 500 h, and self-charges from 0.79 to 0.94 V.

The practical cell viability is dependent on understanding the driving forces in a working battery, and in this electrode less Na^+^ ion battery.

### 2.1. Characterization of Inconel 625 Surface by SKP

**Figure 1:**
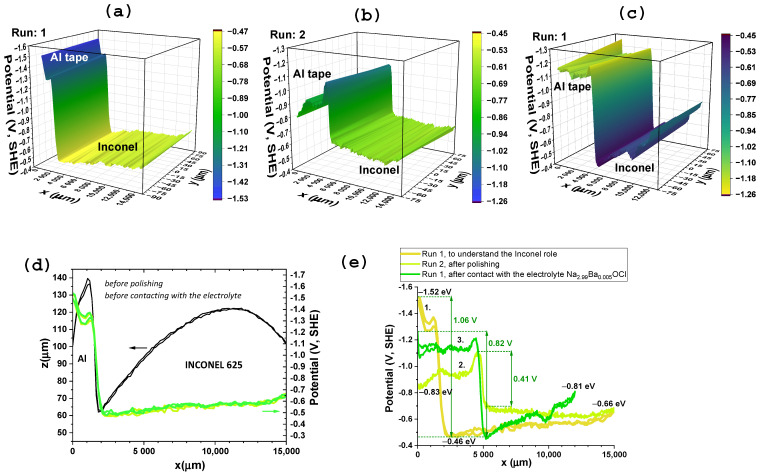
SKP surface potential maps for the Inconel 625 component, using aluminum tape as the reference electrode (negative CC) for: (**a**) 3D plot run 1 to understand the Inconel 625’s role as electrode in the cell and (**b**) 3D plot run 2 after polishing the Inconel before cell assembly; along the x-axis, the region from 0 to 5000 μm (0 to 5 mm) corresponds to the aluminum tape, while the region from 5000 to 15000 μm corresponds to the Inconel 625 surface; (**c**) run 1 after contact with the electrolyte Na_2.99_Ba_0.005_OCl; (**d**) run 1 to understand the Inconel role, 1D topographical and chemical potential analyses of Inconel 625 and aluminum tape demonstrating a difference of 1.09 V corresponding to the open circuit voltage (OCV), and a stable chemical potential (not denoting any charge accumulation) for the Inconel 625 between −0.46 and −0.66 eV; (**e**) differences between chemical potentials (OCV) of Al and Inconel 625 for the two runs prior to contact with the electrolyte and one run after the electrochemical analyses that lasted one year.

SKP measurements were performed with the aim of identifying the positive and negative CC between Inconel 625, Al, Zn and Cu metals. SKP was implemented by opening the sub-circuit on the device and closing the circuit connected to the SKP system, while simultaneously mapping the surface’s capacitive topography and contact potential difference. The aluminum was determined by SKP to be serving as the negative CC. An aluminum tape was used as the reference electrode and the measured contact potential difference ranged from –1.52 V (before) to –0.83 V (after one-year electrochemical cycling) (Figure 1).

In the initial SKP analysis—run 1, it is demonstrated that the potential of the evaluated aluminum tape $\mu_{Al,run1}=-1.52$ eV vs. SHE is similar to the tabled which is $\mu_{Al}=-1.66$ eV vs. SHE [63]; the Inconel 625 chemical potential, to the best of our knowledge, was here determined for the first time as $\mu_{Inconel}\in [-0.66,-0.46]$ eV vs. SHE (Figure 1). The potential difference between Al//Inconel, initially 1.06 V, is achieved along most of the one year of experiments, whenever the constant resistor was not too small (≥5.64 kΩ).

We highlight that the surface potential ϕ difference between the tip and sample, ${\bar{\mu }}_{sample}-{\bar{\mu }}_{tip}=0=\mu_{sample}-\mu_{tip}+ze(\phi_{tip}-\phi_{sample})$, corresponds to the difference in chemical potentials, $∆\mu =\mu_{sample}-\mu_{tip}$ and ultimately allows the calculation of the chemical potential of the $\mu_{sample}$ as the $\mu_{tip}=\mu_{W}=-4.44\;eV$ [64].

Inconel 625’s chemical potential is much less negative than the aluminum reference. The potential of the nickel (Ni^2+^/Ni^0^) $\mu_{Ni}=-0.25$ eV vs. SHE, chromium (Cr^3+^/Cr^0^) $\mu_{Cr}=-0.74$ eV vs. SHE, molybdenum (Mo^3+^/Mo^0^) $\mu_{Mo}=-0.20$ eV vs. SHE, iron (Fe^2+^/Fe^0^) $\mu_{Fe}=-0.44$ eV vs. SHE, aluminum (Al^3+^/Al^0^) $\mu_{Al}=-1.66$ eV vs. SHE, silicon (n-type Si) $\mu_{Si}\approx -0.24$ eV vs. SHE, and niobium (Nb^3+^/Nb^0^) $\mu_{Nb}\approx +1.1$ eV vs. SHE. Considering the relative percentages of the elements, the $\mu_{Inconel}=-0.37=-0.46\pm 0.09$ eV (Ni_41_Cr_15_Mo_3_Fe_2_AlSiNb). This result indicates that the component could function effectively as the positive electrode/current collector, paired with aluminum serving as the negative electrode/current collector $\mu_{Al}=-1.66$ eV vs. SHE. Based on this evidence, the device used in this work was designed considering this electrochemical configuration.

Additionally, to ensure a reliable electrical connection in the battery cell, a copper foil was attached to the Inconel 625 piece using copper tape, providing a conductive interface for the external connection, where alligator clips were later connected. The thin foil does not affect the chemical potential of the solid Inconel as shown in electrochemical cycles.

Curiously, just before contacting with the electrolyte and being introduced in the cell, the Al tape in contact with the Inconel could, after polishing, equilibrate the electrochemical potentials to a difference of 0.41 V much smaller than the initial 1.06 V (Figure 1c). After one year and all the electrochemical cycles, the Inconel equilibrates chemical potentials with the Al away from the interface. The chemical potential assumed by the Al before assembling the cell, after polishing (−0.83 eV), remains as a “memory” and seems to create a target for the charge dynamics of the Inconel in an Al/Na_2.99_Ba_0.005_OCl/Inconel 625 which achieves (−0.81 eV) (Figure 1c). Similar trends were observed before with cells containing other electrolytes, sulfides, oxides, among other types of materials that can align theirs with the electrodes’ chemical potentials away from the interface.

After one year of electrochemical testing, the maximum potential difference between the Al and the Inconel, obtained by SKP, is 0.82 V.

### 2.2. First Principles Simulations for INCONEL625: Electrical, Thermal, and Potential Properties

**Figure 2:**
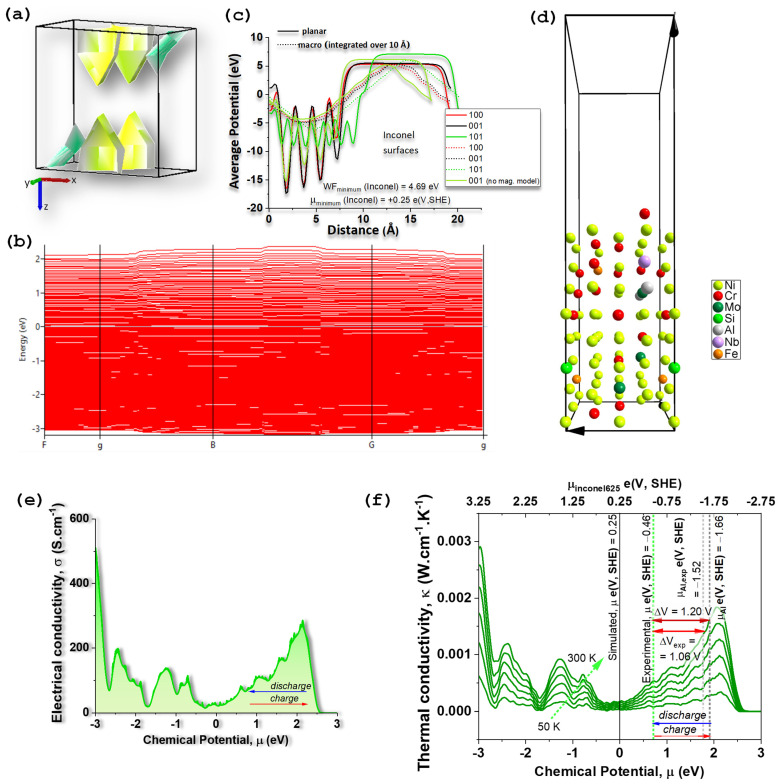
Electric and potential properties of Inconel 625, Ni_41_Cr_15_Mo_3_Fe_2_AlSiNb. (**a**) Fermi surface; the Brillouin zone (first zone of reciprocal space) of the disordered Ni_41_Cr_15_Mo_3_Fe_2_AlSiNb, triclinic P1; yellow/green “surfaces” are the constant-energy contours $E\left(k\right)=E_{F}$; complex shapes indicate multiple bands crossing $E_{F}$ (typical for alloys such as Inconel) and that are shown in (**b**) electronic band structure; (**c**) simulated chemical potential of Inconel showing that the chemical potential of Inconel 625 (Ni_41_Cr_15_Mo_3_Fe_2_AlSiNb) depends on the surface and on the composition of the simulated structure; the minimum work function is WF = 4.69 eV obtained for a (001) surface simulated from a structure optimized not using the magnetic model, corresponding to a minimum chemical potential on the standard hydrogen electrode (SHE), maximum in the absolute scale, where the zero of SHE is $\mu_{0}\left(SHE\right)=-4.44eV$ and 0 eV corresponds to the electrons at rest in a vacuum; (**d**) Inconel 625 surface (010) corresponding to one of the simulated surfaces used for the calculation of the work function; (**e**) electrical conductivity of the Inconel 625 while charging and discharging it; (**f**) thermal conductivity of the Inconel 625 while charging and discharging it; potential difference between Inconel and Al corresponding to the open circuit voltage of an All//Inconel 625 cell; the SHE scale for the thermal conductivity serves equally for the electrical conductivity in (**e**).

The first-principles results rationalize the experimentally observed behavior of the Al/Na_2.99_Ba_0.005_OCl/Inconel 625 device and clarify the role of Inconel as an electronically conductive, electrochemically inert CC whose surface potential sets the positive electrode’s chemical potential [65]. The ordered supercell chosen to represent Inconel 625 (Ni_41_Cr_15_Mo_3_Fe_2_AlSiNb; 64 atoms) relaxes to an fcc-A1, Fm-3m, Ni–based solid solution. It exhibits a metallic band structure with multiple bands crossing the Fermi level E_F_ and a complex Fermi surface (Figure 2a) consistent with a high density of states at E_F_ (Figure 2b) and elevated electrical conductivity required of a CC (Figure 2e).

A key outcome is the surface-dependent work function (WF) WF $=-\bar{\mu }$ (on the vacuum scale) and corresponding electron chemical potential μ (Figure 2c). Among the low-index simulated surfaces (Figure 2c,d), the minimum work function is ~4.9 eV (vacuum at 0 eV), which corresponds to a chemical potential $\mu_{Inconel\;625}$ ≈ +0.26 e(V vs. SHE). This value is in acceptable agreement with SKP measurements that place Inconel’s chemical potential in the interval $\mu_{Inconel\;625}$ ∈ [−0.66,−0.46] e(V vs. SHE) before and after a year-long operation. $\mu_{Inconel\;625}$ ≈ +0.26 e(V vs. SHE) is in better agreement with the value obtained from the relative amount of the elements $\mu_{Inconel\;625}$ ≈ −0.37 e(V vs. SHE) (Ni_41_Cr_15_Mo_3_Fe_2_AlSiNb). This concordance indicates that ***(i)*** the alloy’s surface energetics is captured at the right magnitude by the model, and ***(ii)*** the Inconel surface remains chemically and electronically stable during cycling—a conclusion independently supported by SEM/EDX.

Using the simulated surface chemical potentials, the predicted open-circuit voltage (OCV) for Al//Inconel625 follows directly from the difference in chemical potentials Δμ between the two metals. The measured Inconel 625 (Ni_41_Cr_15_Mo_3_Fe_2_AlSiNb) value together with the measured and tabulated Al [63] potential yields ≈ 1.1–1.2 V, matching the observed long, flat discharge plateau near 1.0–1.05 V across widely different loads and test histories. The small OCV decay after extended operation (to ~0.93 V) tracks the gradual rise in interfacial resistance extracted from impedance fits (growth of R3) and the emergence of Al(OH)_3_ signatures detected by XRD as shown hereafter, pointing to interfacial—not bulk—origins for the voltage loss. Thus, the simulations support the interpretation that the device’s working voltage is set by the metals’ surface chemical potential difference, while capacity and ageing are governed by electrolyte/electrode double-layer formation [65] and the stability of the Al interface. In other words, the potential difference in a battery depends on the difference in chemical potentials of the materials in contact with the electrolyte [65].

The calculated electronic structure (Figure 2e) also explains why Inconel remains effectively “capacity-neutral”. In the ordered cell, spin-polarized calculations yield a half-metallic ferromagnetic ground state with a small total moment (11.95 μB per 64-atom supercell). In a real, compositionally disordered alloy, long-range ferromagnetism is not expected; the simulated moment likely reflects the artificial ordering of solutes in a finite cell. Crucially, this does not undermine the work function (Figure 2c,d) trends or the conclusion that no faradaic Na^+^ uptake occurs in Inconel under the present conditions—consistent with SKP stability and the absence of compositional change by EDX.

Finally, the transport trends (Figure 2e,f) inferred from the simulations (metallic conductivity σ with weak bias dependence; thermal conductivity κ consistent with a dense Ni-rich alloy) align with impedance evidence that the bulk of the CC does not limit performance; rather, the governing elements are the electrolyte’s bulk response (first semicircle) and the two EDLC-dominated interfaces (additional semicircles), which evolve with cycling/history, as shown hereafter.

Together, the DFT results and surface energetics provide a quantitative foundation for materials selection: a positive CC should maximize the WF (to raise ΔV vs. Al), remain chemically inert to Na^+^/OH^−^/Cl^−^/NaO^−^ species, and present stable, clean surfaces—criteria that Inconel 625 meets under our processing and operating window. Future simulation refinements (larger special-quasi-random supercells, stricter k-point/energy convergence, explicit electrolyte/metal surfaces) can refine the spread of WF across surfaces, but are unlikely to change the central result: ***(i)*** the Al//Inconel voltage is a thermodynamic property set by the metals’ surface chemical potentials, and ***(ii)*** Inconel’s chemical potential remains within the narrow range measured by SKP throughout long-term operation, as shown hereafter.

### 2.3. Electrochemical Performance of the Device

**Figure 3:**
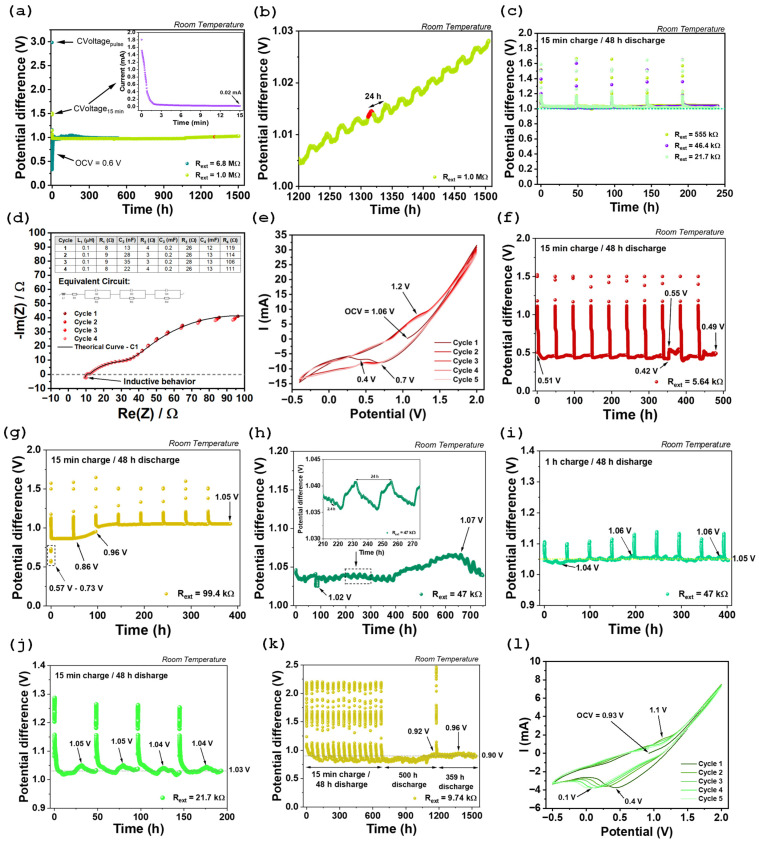
Potential difference measurements over time under different external resistors loads: (**a**) device response with 6.8 MΩ and 1 MΩ resistors connected across the terminals; (**b**) zoom-in of the voltage profile under 1 MΩ between 1200 and 1500 h, highlighting long-period oscillations; (**c**) potential difference evolution under external resistors of 555 kΩ, 46.4 kΩ, and 21.7 kΩ. Electrochemical characterization of the device following charge/discharge cycling under a 21.7 Ω external resistor load: (**d**) Nyquist plot from PEIS analysis; (**e**) cyclic voltammetry curve. Potential difference measurements over time under different external resistors: (**f**) cell response for 5.64 kΩ; (**g**) cell response for 99.4 kΩ; (**h**) continuous discharge with a 47 kΩ resistor, without preliminary charging; (**i**) 1 h charge at 1.1 V followed by a 48 h discharge step. (**j**) cell response for 21.7 kΩ; (**k**) cell response for 9.74 kΩ; (**l**) CV curve performed after (**k**). Note: efficient surface area ~15 cm^2^.

The study initiated with an OCV measurement, which yielded 0.6 V. This measurement intended to assess the initial state of the cell and estimate its operating range. The device was then subjected to a prolonged discharge phase under high external resistances (6.8 MΩ and 1 MΩ) connected across its terminals. This configuration closely approximates open-circuit conditions and was employed to evaluate the maximum potential the system could sustain over time (Figure 3a,b). The experimental procedure involved applying a short constant voltage (CVoltage) pulse at 3 V for one second, using a 6.8 MΩ input resistance (see dark green data in Figure 3a). This step was immediately followed by an extended discharge period through the 6.8 MΩ resistor lasting approximately 532 h, during which a voltage of approximately 1 V was observed. Subsequently, the external resistance was reduced to 1 MΩ Figure 3a,b, and a CVoltage step at 1.5 V was applied for 15 min to monitor the evolution of the current over this interval (inset of Figure 3a). The initial current measured was 1.8 mA, which gradually declined to 0.02 mA by the end of the charging period (Figure 3a). During the discharge phase with 1 MΩ resistance, a potential difference of ~1 V was maintained for about 1500 h. A detailed examination of the final 300 h reveals a clear oscillatory pattern with a period of roughly 24 h, superimposed on a steadily increasing voltage trend (Figure 3b).

Subsequently, the external resistors were replaced with significantly lower values, namely 555 kΩ, 46.4 kΩ, and 21.7 Ω (Figure 3c). In this set of experiments, a protocol was followed consisting of 15 min of charging in CVoltage mode, followed by a 48 h discharge, during which potential differences above 1 V were observed (Figure 3c).

The results presented in Figure 3c were analyzed in detail, and the calculated discharge currents and capacities are summarized in Table A1 of the Appendix A. It is worth noting that the average voltages obtained are very similar for the resistors of 555 kΩ, 46.4 kΩ, and 21.7 kΩ. The average discharge currents over five cycles increase accordingly, from 2 to 22 to 48 μA, respectively. Consequently, the corresponding discharge capacities increase from 0.089 to 1.061 to 2.268 mA.h, respectively. The latest of which corresponds to 0.151 mAh.cm^−2^.

**Figure 4:**
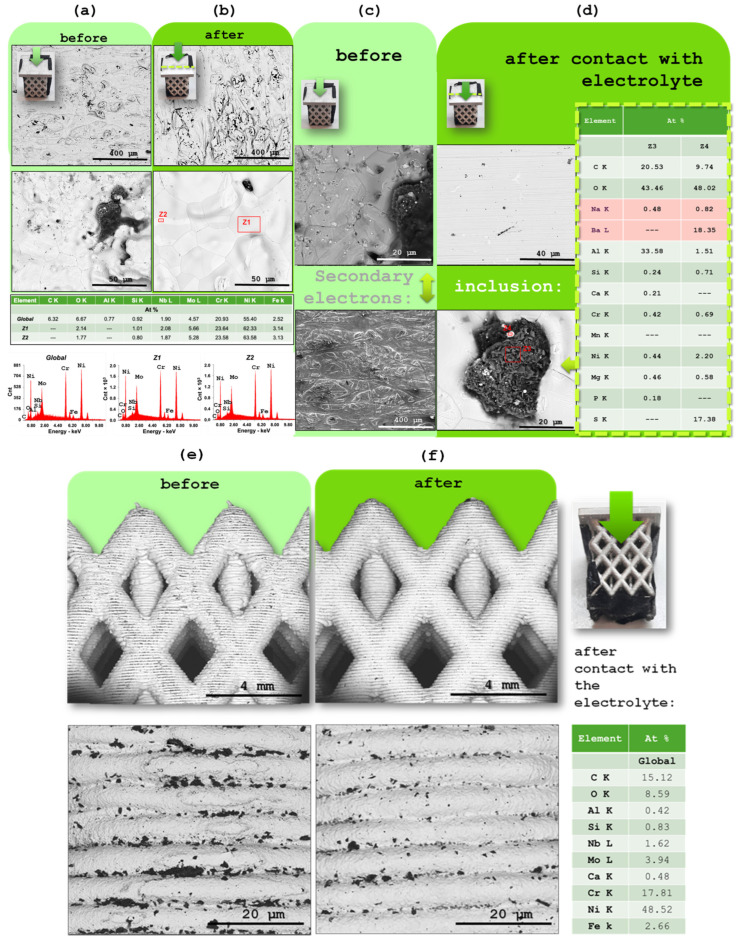
Characterization of Inconel 625 flat surface by SEM/EDX using a backscattered electron (BSE) detector: (**a**) pristine sample with no contact with the electrolyte, at 250× and 2000× magnifications; (**b**) sample after electrochemical experiments at 250× and 2000× magnifications; (**c**) pristine sample with no contact with the electrolyte, at 2000× and 250× magnification (secondary electrons SE); (**d**) Inconel 625 after electrochemical experiment, at 2000× magnification. EDX spectrum of the red-marked regions shown in (**b**) and (**d**) inclusion acquired with an electron beam energy of 15 keV. Characterization of the interlaced lateral area of Inconel 625 by SEM/EDX using a backscattered electron (BSE) detector: (**e**) pristine sample with no contact with the electrolyte, at 25× and 250× magnification, respectively; and (**f**) sample after electrochemical experiments at 25× and 250× magnification, respectively. Right, EDX analysis of the Inconel interlaced lateral area after electrochemical analyses.

The relatively stable average potential difference under different discharge conditions indicates good voltage regulation and high electrochemical stability of the system, which is promising for practical energy storage applications. After the discharge under an external resistor of 21.7 kΩ, potentiostatic electrochemical impedance spectroscopy (PEIS) was performed, followed by cyclic voltammetry (CV) (Figure 4d,e).

The PEIS data were subsequently fitted to an equivalent circuit model (Figure 3d). The Nyquist plots provide detailed insights into the electrochemical behavior of the device-cell, including the bulk electrolyte resistance, which is directly related to its ionic conductivity. Additionally, the charge transfer resistance at the electrolyte/CC interface (Inconel 625 or Al) can be assessed. PEIS also enables the estimation of the double-layer capacitance, which reflects the charge storage capacity at the interfaces.

At higher frequencies in the impedance spectroscopy measurements, a negative imaginary component of the impedance was observed, indicating inductive behavior. This effect may be attributed to extremely fast relaxation processes associated with freer ions in certain regions in the solid electrolyte. These regions can exhibit distinct polarization dynamics and responses to the alternating electric field, resulting in a phase shift characteristic of an effective inductive response. Such behavior has been reported in complex ionic materials, where structural heterogeneities or local electromechanical couplings induce particularly rapid transient responses [66,67]. The presence of this effect in the high-frequency regime may therefore provide additional insight into the microstructure and dynamic internal properties of the electrolyte.

Following this initial high-frequency inductive behavior (L_1_), a well-defined semicircle corresponding to C_2_ and R_2_ is observed in the Nyquist plot, corresponding to the ionic transport within the bulk of the electrolyte. This feature typically appears at high frequencies and reflects the dominant contribution of the mobile ionic species to the overall conductivity. In this system, the Na^+^ ion is the primary charge carrier, and its motion through the crystalline/amorphous matrix of Na_2.99_Ba_0.005_ClO gives rise to the characteristic impedance response.

The highest-frequency semicircle observed in the Nyquist plot should be modeled using an equivalent circuit consisting of a resistor R_2_ in parallel with a capacitor C_2_, both in series with a resistor R_1_ (Figure 3d). In this model, R_2_ represents the ionic resistance associated with the bulk transport of Na^+^ ions through the electrolyte, while hopping under alternate current (AC) in a region where the ions move more freely and are not significantly influenced by Coulombic interactions arising from charge accumulation at the interfaces. This resistance is therefore directly related to the intrinsic ionic conductivity of the electrolyte material. In contrast, R_1_ represents electrical and ionic resistances that occur independently at each interface or for each ion species in the cell. In other words, these are local resistances that affect only one surface or interface and do not impact the electrolyte and both electrode surfaces simultaneously.

Additional semicircles observed at lower frequencies correspond to interfacial phenomena, such as the spontaneous formation of electrical double-layer capacitors (EDLCs), which compensate for differences in chemical potential between the electrolyte and the electrodes, as mentioned before. The second semicircle can be modeled as a parallel association of capacitor C_3_ and resistor R_3_, representing the electrolyte/electrode interfacial EDLC. A third semicircle, represented by resistor R_4_ in parallel with capacitor C_4_, likely corresponds to the resistance to ionic movement within an EDLC at another interface of the Inconel/Na_2.99_Ba_0.005_ClO/Al battery cell. These EDLCs form spontaneously to balance the electrochemical potentials between the electrolyte and the electrodes and CC. We highlight that the electrochemical potentials may not be equalized by electron tunneling and conduction to the bulk electrolyte as the electrolyte is an insulator.

Approximating the square cross section to circular solid coaxial cell, the cross-sectional resistance (“radial resistance”) [68],

(1) $$R=\frac{1}{2\pi \sigma d}ln\frac{b}{a}$$

where $R=R_{2}$ is the coaxial cell resistance to ion hopping from a to b within the bulk electrolyte, d the length of the cell, a the inner radius and b the outer radius of the electrolyte. Attending the cells’ dimensions and to Equation (1): a=1.37 cm, b=2.185 cm, d=2.12 cm; ${<R}_{2}>=3.5$ Ω (Figure 3d) and σ=10 mS.cm^−1^.

Using the same approaches, we have calculated the dielectric constant $\varepsilon_{r}$ of the electrolyte using Equation (2) [68],

(2) $$C=\frac{2\pi \varepsilon_{0}\varepsilon_{r}d}{ln\frac{b}{a}}$$

where ${<C}_{2}>=24.5$ nF (Figure 3d) and $\varepsilon_{0}=8.85\times 10^{-12}$ Fm^−1^, which yields $\varepsilon_{r}\approx 9706\approx 10^{4}$, at room temperature. This analysis enables a quantitative evaluation of the bulk ionic transport properties although it holds an approximation essential for assessing the electrolyte’s performance in solid-state devices. This approximation does not account for the potential accumulation of charge at the square corners.

Cyclic voltammetry (CV) enables the analysis of charge transfer kinetics and the identification of multistep redox processes, as well as distinguishing between capacitive and pseudocapacitive charge storage mechanisms. In this work, CV measurements were performed within a voltage window of −0.4 to 2.0 V (Figure 3e). Redox peaks were observed at 1.2 V while charging, and at 0.7 and 0.4 V while discharging, which are associated with electron tunneling events leading to Na^0^ plating. The OCV was approximately 1.06 V, in agreement with the theoretical value derived from the difference in chemical potentials of the CC and in agreement with SKP data, Figure 1 and Figure 2,

(3) $$OCV=\frac{\mu_{Al}-\mu_{Inconel}}{e}\approx 1.1\;V$$

While charging above 1.1 V, Al is reduced and Inconel oxidized, and while discharging below 1.1 V, Inconel is reduced and Al oxidized. This confirms the active electrical role -while not chemical—of the CC in the absence of conventional redox electrodes.

After evaluating the intrinsic electrochemical behavior of the device through PEIS and CV, the next step involved connecting an external resistor of approximately 5.64 kΩ in parallel across the device terminals (Figure 3f). Interestingly, even before charging, a measurable voltage of approximately 0.51 V was observed across the terminals. This voltage was maintained during the first minute after the resistor had been connected, indicating that the device retained a significant amount of stored charge from its initial state. The same protocol previously described was then applied: 15 min of constant voltage charging at 1.5 V followed by 48 h of discharge (Table A1 of the Appendix A). A total of 10 cycles were performed, and the device exhibited highly consistent behavior across cycles, except for cycle 8. In this case, two distinct self-charging events were observed during the discharge period, the most pronounced of which showed a voltage increase from 0.42 to 0.55 V.

However, since the discharge plateau remained well below the natural OCV, the external resistance was significantly increased to 99.4 kΩ to investigate the device’s response under a lower discharge current (Figure 3g and Table A1 of the Appendix A). Before applying the charging step, the open-circuit voltage was monitored for 10 min with the resistance connected. During this period, a progressive increase in potential difference was observed, rising from 0.57 to 0.73 V, indicating spontaneous self-charging behavior. Following this observation, eight charge/discharge cycles were performed. Notably, in the second cycle, a substantial voltage increase was observed during the discharge phase (from 0.86 to 0.96 V), after which the device stabilized at a discharge plateau of approximately 1.05 V over the following five consecutive cycles.

After confirming that the device operated effectively under a 99.4 kΩ load, the external resistor was reduced to approximately half (47 kΩ), and the system was left in continuous discharge mode without any external charging input (Figure 3h). Remarkably, the device maintained a terminal voltage above 1.02 V for approximately 750 h, despite the continuous discharge through the external resistor, which remained connected in parallel across the terminals. Two distinct self-charging events were observed during this period (detail view of Figure 3h). One of these occurred over a 24 h interval, while the second, less pronounced event, spanned approximately 2.4 h.

Following these results and keeping the same external resistor (47 kΩ), the device was charged for 1 h in CV mode at 1.1 V, followed by a 48-h discharge period (Figure 3h). This procedure aimed to assess whether the discharge plateaus voltage could be further elevated allowing a direct comparison with previous results presented in Figure 3c (46.4 kΩ) and Table A1 of the Appendix A. The results clearly demonstrate the occurrence of multiple self-charging events during the discharge phases. The average voltage during discharge was approximately 1.05 V, which closely matches or even surpasses the value obtained in earlier tests, confirming that the device continued to operate reliably.

The study proceeded with a reduction in the external resistance to 21.7 kΩ (Figure 3j), and then to 9.74 kΩ (Figure 3k). In Figure 3k, four consecutive charge/discharge cycles were performed under an external resistance of 21.7 kΩ, with an average voltage of approximately 1.03 V recorded during the discharge steps (Table A1 of the Appendix A). These results demonstrate good reliability in the cell’s performance and are very similar to those previously obtained, as shown in Figure 3c and Table A1 of the Appendix A. This consistency reinforces the electrochemical system’s stability over time.

Regarding the set of experiments carried out with an external resistance of 9.74 kΩ, a profile comprising 14 charge/discharge cycles was recorded (Figure 3k), where the discharge plateaus were observed to remain below 0.9 V. Subsequently, a 500 h discharge-only run was conducted, during which the voltage increased to approximately 0.92 V near the end of the discharge period. Finally, after a brief 15 min charging step between the two long discharge phases, another discharge stage lasting around 359 h was performed, with the voltage plateau stabilizing at approximately 0.90 V. As expected, the results obtained were better than those recorded with a resistance of 5.64 kΩ (Figure 3f) but worse than those with 21.7 kΩ (Figure 3c,j), although the system remained stable over time.

**Figure A1:**
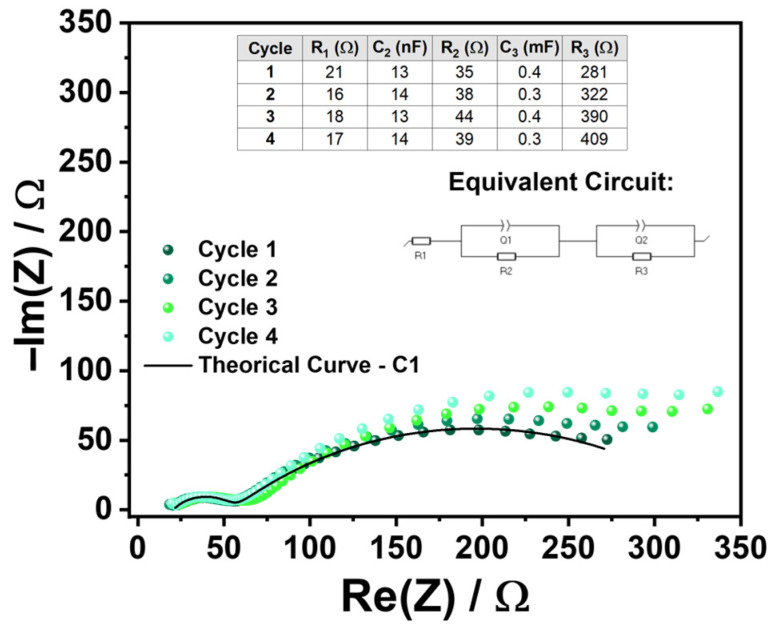
PEIS analysis and Nyquist plot after charge/discharge cycling under a 9.74 kΩ external resistor, after approximately one year from battery-cell fabrication.

New PEIS and CV analyses (Figure A1 of the Appendix A and Figure 3l) were performed at this stage to monitor the electrochemical evolution of the device and compare it with earlier results (Figure 3d,e), particularly regarding internal resistance and redox activity.

Regarding the PEIS results, a general analysis reveals that the inductive behavior previously observed at higher frequencies is no longer present, and only two semicircles are now visible. A more detailed comparison of the data presented in the corresponding figures (Figure 3d and Figure A1 of the Appendix A) shows that the resistances increased significantly, particularly R_2_ and R_3_. Additionally, in the latest measurements (Figure 3l), the five cycles display overlapping responses at higher frequencies (corresponding to the first semicircle), indicating stability in that region. However, as the experiment progressed, a gradual increase in R_3_ was observed, suggesting interfacial degradation or the accumulation of resistive by-products at the electrode/electrolyte interface. These changes may be associated with slow kinetic processes such as ion trapping, interphase layer formation (Na_2_O or Al(OH)_3_), or limited ion mobility during extended discharge periods.

Regarding the CV results (Figure 3l), it is immediately apparent that, compared to the previously obtained data (Figure 3e), it was not possible to reach current values as high for the same potential range, which likely indicates a decrease in the system’s ionic and/or electronic conductivity. This reduction may be associated with degradation at the electrode/electrolyte interfaces, the formation of passivating layers that hinder charge transfer, or structural changes in the active materials caused by prolonged discharge periods. Additionally, it should be noted that the system began this analysis with a lower OCV of 0.93 V (very similar to the last discharge voltage 0.90 V), and a slight peak can be observed around 1.1 V, as well as a corresponding response in the reverse scan between 0.4 and 0.1 V, suggesting the presence of low-intensity reversible redox processes, which may include electron tunneling to the surface of the electrolyte and possibly Na^0^ plating.

In summary, the electrochemical analysis provided important insights into the device’s performance and its evolution under different external resistances. However, to better understand the physical and chemical state of the CC after the electrochemical tests, the device was disassembled to allow direct examination of the Inconel 625 and aluminum components. Post-mortem SKP results obtained from the Inconel 625 and Al pieces after disassembly, and complementary SEM/EDX and XRD analyses aimed at identifying possible material changes occurring during operation.

### 2.4. SKP Evaluation of Inconel 625 Surface After Electrolyte Contact

The SKP analyses were performed after device extended electrochemical testing, which included PEIS, CV, and charge/discharge cycles under different discharge currents. The device remained assembled and under testing conditions for approximately one year. After this period, the Inconel 625 component was re-analyzed using SKP under a configuration like the initial setup, employing an aluminum tape as the reference electrode (Figure 1c,e).

The surface potential of Inconel 625 remained within the range of −0.46 to −81 V even after prolonged operation (Figure A1 of the Appendix A), showing no significant electrochemical degradation compared to the initial measurements (Figure 1c,e). This stability, despite extended contact with a sodium-based ferroionic electrolyte (Na_2.99_Ba_0.005_OCl) known to be chemically reactive with certain metals, highlights the electrochemical robustness of Inconel 625 under the tested conditions and supports its suitability as a durable CC in long-term electrochemical systems.

### 2.5. SEM/EDX Characterization

In this section, both the Inconel 625 and aluminum CC were analyzed by scanning electron microscopy (SEM) and energy-dispersive X-ray spectroscopy (EDX), with the aim of assessing possible morphological and compositional changes resulting from contact with the solid electrolyte. For this purpose, Inconel 625 samples were collected in both their pristine state (prior to any exposure to the electrolyte) and after disassembly of the device following charge/discharge cycles (Figure 4a–f). They show almost no difference and no significant presence of sodium Figure 4d.

The interlaced lateral area of the Inconel 625 piece was also analyzed by SEM (Figure 4e,f). Even in this region, where contact with the electrolyte occurred over a larger surface area, no visible signs of degradation were identified. The surface morphology remains stable, indicating that the material retains its integrity even under conditions of increased exposure to the Na^+^-based electrolyte.

**Figure 5:**
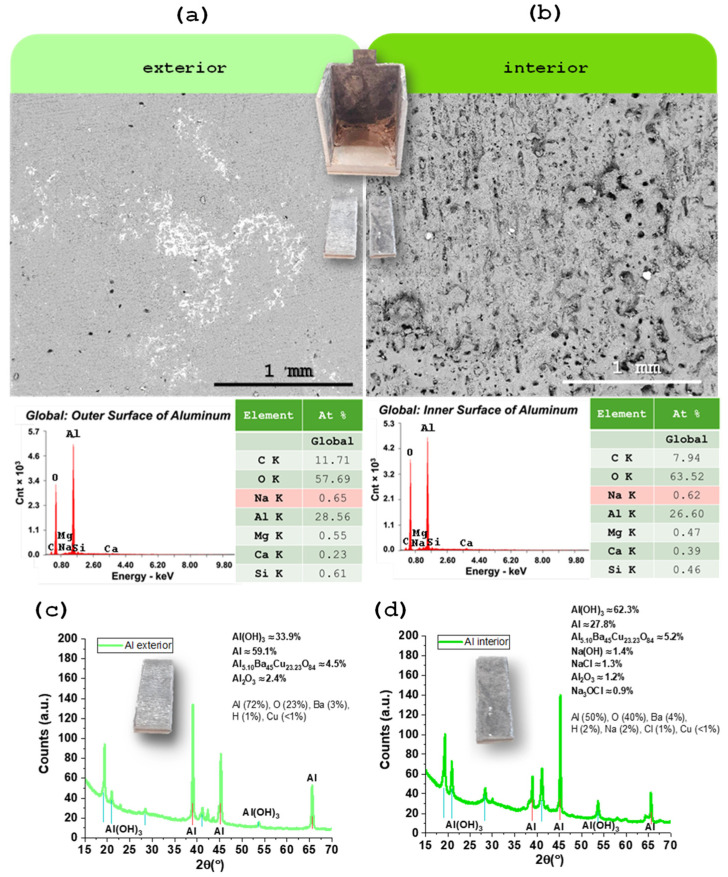
Characterization of the aluminum CC by SEM/EDX: (**a**) SEM image of the external surface; (**b**) SEM image of the internal surface (in contact with the electrolyte). EDX spectrum of the outer and inner surfaces. Characterization of the aluminum CC by XRD: (**c**) XRD diffractogram of the external surface; (**d**) XRD diffractogram of the internal surface (in contact with the electrolyte). The main difference between the two samples is that the internal surface is more oxidized than the external, likely through a raise in Al(OH)_3_ in detriment of pure Al. The dry electrolyte, as shown by SEM/EDX, does not react with the Al. Barium from the electrolyte is likely to accumulate on the negative electrode interface, in agreement with these results that indicate that an Al-Ba-Cu oxide may be present.

Aluminum samples were taken from the external surface (not in contact with the electrolyte) and the internal surface (which had direct contact with the electrolyte) (Figure 5a,b). SEM analysis was performed to allow a detailed microscopic comparison of the surface features (Figure 5a,b). These observations were complemented by EDX measurements to investigate potential chemical changes or the formation of surface layers during cell operation (Figure 5a,b).

By comparing the SEM images of the Inconel 625 samples before and after contact with the electrolyte upon device operation, it is observed that the surface morphology remains practically unchanged (Figure 4a–f). No signs of degradation or structural alterations were detected on the flat area, indicating good chemical and mechanical stability of the material. This conclusion is further supported by the EDX analysis, which shows no significant signs of oxidation or changes in the composition of the Inconel 625. These results confirm that Inconel 625 preserves its integrity even after prolonged electrochemical testing, demonstrating its suitability as a CC in this system.

In the case of aluminum, used as the negative CC, it was immediately observed after the disassembly of the device that the surface in contact with the electrolyte (inner surface) showed visible signs of degradation, including noticeable roughness visible to the naked eye (Figure 5b). However, EDX analysis revealed similar results for both the inner and outer surfaces, suggesting that despite the evident mechanical degradation, no significant changes occurred in the chemical composition of the material (Figure 5a,b). This indicates that aluminum maintains satisfactory electrochemical stability during device operation. XRD later revealed an increase in Al(OH)_3_ in detriment of Al. The observed structural degradation due to moisture residue may represent a potential point of failure in long-term use.

### 2.6. XRD Characterization of the Aluminum

X-ray diffraction was used to probe phase changes on the aluminum CC after one year of operation (Figure 5c,d), comparing cut pieces from the outer surface (no electrolyte contact), Figure 5c, and inner surface (in contact with Na_2.99_Ba_0.005_OCl), Figure 5d.

The outer Al surface diffractogram is dominated by metallic Al reflections, consistent with a largely unaltered fcc aluminum substrate. In contrast, the inner surface shows a clear increase in oxide/hydroxide signatures, most notably peaks assignable to Al(OH)_3_, alongside a relative reduction in the metallic Al peak intensity. This points to preferential surface oxidation/hydroxylation on the electrolyte-facing side during service.

These XRD trends align with the complementary microscopy: SEM visually reveals roughening of the inner Al surface, while EDX shows no gross compositional shift at the micron scale—i.e., oxidation is confined to a thin surface region that XRD detects sensitively, but which does not substantially change the bulk stoichiometry sampled by EDX. The dry electrolyte itself does not react with Al in bulk; instead, the XRD difference is consistent with surface layer growth under operating conditions.

**Figure 6:**
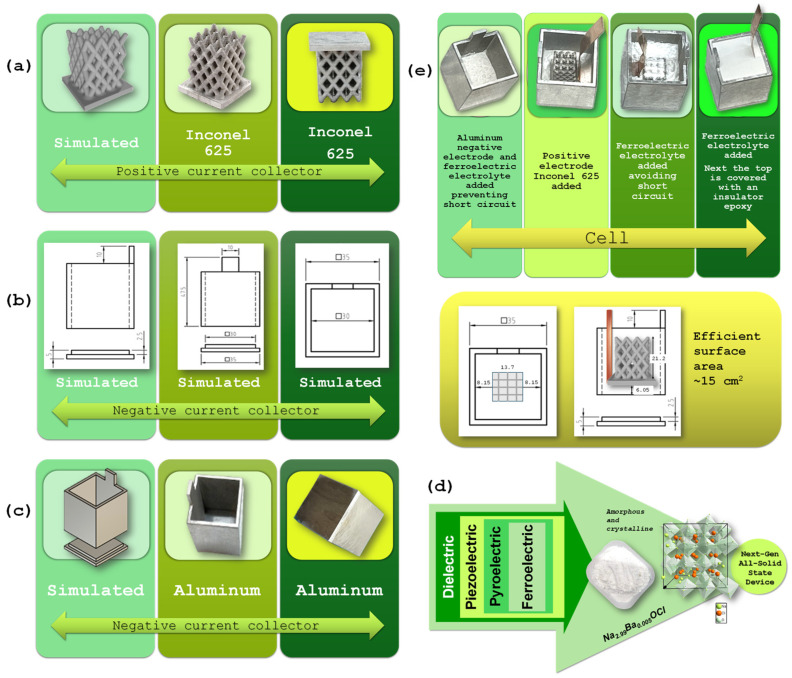
Development of the Inconel 625 component: (**a**) 3D models designed in SolidWorks (simulated); and final part obtained after the FFF printing and sintering process; (**b**,**c**) design and fabrication of the aluminum container: (**b**) CAD models developed in SolidWorks; and (**c**) 3D simulation and CNC (computer numerical control)-machined aluminum components; (**d**) Schematic representation of the Na_2.99_Ba_0.005_OCl solid electrolyte, displaying powder morphology and disordered crystal structure model (generated using Diamond 4.6.8 software). The image conceptually illustrates the relationship between the material’s atomic structure and its multifunctional ferroelectric behavior, supporting its applicability in all-solid-state energy devices; (**e**) Step-by-step of the device assembly process: deposition of the solid electrolyte powder onto the base of the aluminum container; placement of the Inconel 625 component with attached copper foil at the center of the Al container; gradual addition and mechanical compaction of electrolyte powder to infiltrate the porous Inconel structure; and final configuration after complete filling, ensuring uniform electrolyte distribution and intimate contact between components. Dimensions of the components of the cell. Note: measurements are given in millimeters unless stated otherwise.

The data further suggest interfacial speciation at the negative electrode: the authors note that Ba from the electrolyte likely accumulates at the Al interface, and an Al–Ba–Cu oxide may form. The Cu may have been originated on the Cu tab of the Inconel (Figure 6e). Such minor phases—difficult to resolve unambiguously without dedicated reference scans and grazing-incidence geometry—could contribute weak reflections in the inner-surface pattern and are consistent with interfacial chemistry driven by the local field and ionic environment.

Importantly, the electrolyte is amorphous/glassy but may show crystalline phases; a diffuse background from any adherent residue would be more likely than sharp reflections. This matches the absence of new crystalline electrolyte phases in the Al patterns.

Finally, no evidence of phase change was reported for Inconel625, as referred previously; stability of the positive CC is instead corroborated by SEM/EDX and SKP, which show no significant degradation in composition or surface potential after long-term cycling. Thus, the XRD results reinforce a picture in which aging is localized at the Al/electrolyte interface (growth of Al-oxy/hydroxide), while Inconel remains structurally and chemically robust under the tested conditions.

**Figure A2:**
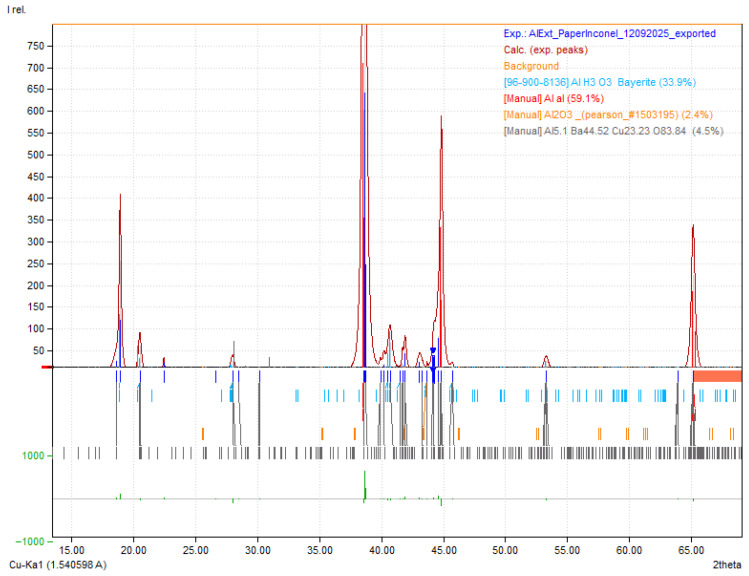
Characterization of the aluminum CC by XRD: XRD diffractogram of the external surface.

**Figure A3:**
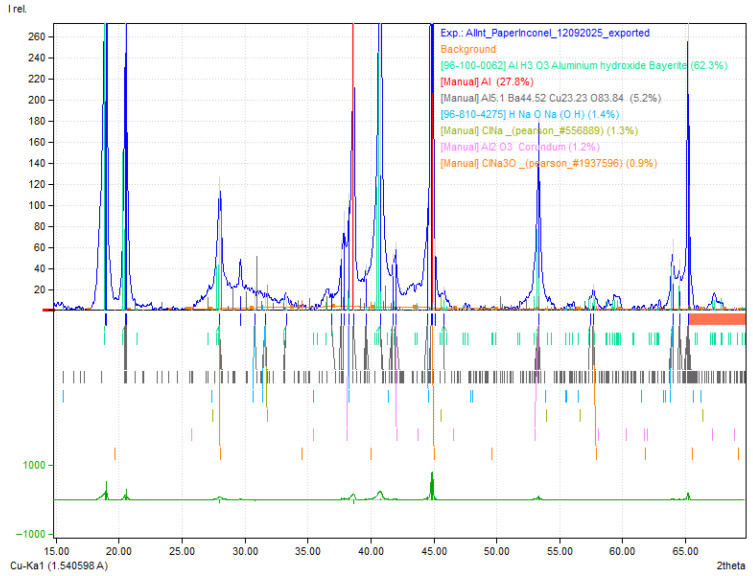
Characterization of the aluminum CC by XRD: XRD diffractogram of the internal surface.

On the XRD pattern of the Al, anisotropy at 38.5–38.6° versus 44.8° (2θ) was detected with the highest peak at 38.60° (2θ) and approximately 140 counts, followed by aluminum hydroxides (Al(OH)_3_) with highest peak at 18.19° (2θ) (Figure A2 and Figure A3 of the Appendix A for matching detail). For more accurate quantitative data Rietveld refinement (RR) would have to be performed. However, the bulk samples were not suitable for RR and a compromise, and careful analyses was established resulting in a coherent outcome.

## 3. Materials and Methods

This section describes the materials used in the fabrication of the energy harvesting and storage battery, namely Inconel 625 (Figure 6a) and an aluminum container (Figure 6b,c), and the solid-state electrolyte Na_2.99_Ba_0.005_OCl (Figure 6d), along with the methodologies applied during device assembly. The experimental procedures adopted for material and interface characterization are also presented, including SKP for surface potential mapping, SEM/EDX and XRD for morphological and compositional analysis, and electrochemical techniques such as PEIS, CV, and charge/discharge cycles to assess electrochemical behavior and device performance.

### 3.1. Inconel 625 and Aluminum Container

The geometry of the CC acting as positive electrode part was designed in a CAD environment using *SolidWorks* 2024 and subsequently exported in STL format, compatible with the 3D manufacturing process (Figure 6a). The STL file was processed using slicing software, where processing parameters such as layer height, infill, and extruder temperature were defined. The part was fabricated via FFF, using a composite filament consisting of Inconel 625 powder dispersed in a polymeric binder. The production component, referred to as the “green part,” underwent thermal debinding to remove the binder, followed by high-temperature sintering, resulting in the consolidation of the metallic material and the attainment of the final properties (Figure 6a).

The aluminum component was designed in a CAD environment using *SolidWorks* (Figure 6b,c). The design was divided into two parts to facilitate the manufacturing process. Both halves were machined from aluminum using CNC (computer numerical control) technology (Figure 6c right). After machining, the parts were bonded together to form a single component, which was then used in the experimental setup as a functional element of the device.

### 3.2. Solid-State Electrolyte: Na_2.99_Ba_0.005_OCl

The electrolyte employed was Na_2.99_Ba_0.005_OCl, a glassy ferroelectric material synthesized via water solvation. Commercial-grade stoichiometric amounts of NaCl (≥99.0%), anhydrous NaOH (≥99%), and anhydrous Ba(OH)_2_ (94–98%) were dissolved in deionized water to obtain a homogeneous aqueous solution. The mixture was thermally dried at 250 °C for 2 h, yielding a partially hydroxylated intermediate (Na_2.99_Ba_0.005_Cl_1-x_O(OH)_x_). A subsequent drying step at >230 °C eliminated residual hydroxyl groups, resulting in the formation of the final glassy Na_2.99_Ba_0.005_OCl phase. The dried solid was then subjected to ball milling in a sealed agate vessel containing five agate balls (20 mm diameter) at 300 rpm for 40 min, ensuring reduced particle size, compositional homogeneity, and limited moisture uptake. The resulting powder was transferred into a glovebox with O_2_ and H_2_O levels maintained below 1 ppm, where it was integrated into the energy harvesting and storage device [56,57].

This electrolyte is a glassy ferroelectric material (Figure 6d), synthesized via a water-mediated method that is both cost-effective and environmentally friendly, relying solely on water as the solvent. This material exhibits ferroelectric behavior and, therefore, belongs to the class of pyroelectric materials, which are a subset of piezoelectric materials, and part of the broader family of dielectric materials. Owing to this combination of functional properties, it holds strong potential for use in next-generation all-solid-state energy devices (Figure 6). Due to its self-charging and self-cycling behavior, it was chosen to understand the behavior of inert electrodes/electrolyte in a battery-cell.

### 3.3. Battery Cell Assembly

**Figure 7:**
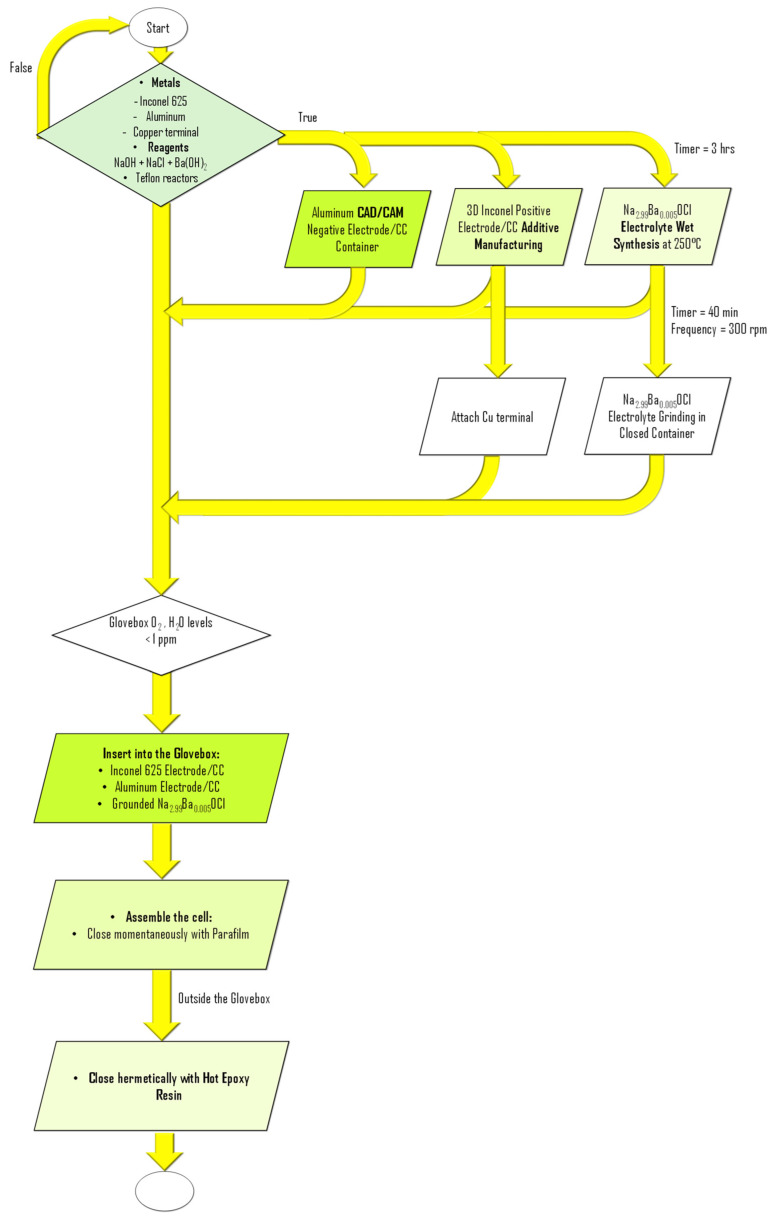
Flowchart for the fabrication of Al/Na_2.99_Ba_0.005_OCl/Inconel 625 battery cell.

Device assembly was performed entirely inside a glovebox maintained with O_2_ and H_2_O levels below 1 ppm (Figure 7). Initially, a copper foil was attached to the Inconel 625 component using copper tape, serving as an electrical connector to the external circuit. The solid electrolyte powder was first deposited onto the base of the aluminum container, forming a compact foundation layer (Figure 6e and Figure 7), avoiding short circuits. The Inconel component with the attached copper foil was then placed at the center of the aluminum container. Additional electrolyte powder was gradually added and mechanically compacted using a spatula to promote infiltration into the porous architecture of the Inconel structure. This process was repeated until the aluminum container was fully filled, ensuring intimate interfacial contact between components and homogeneous distribution of the electrolyte throughout the device (Figure 6e and Figure 7). The cell was then closed with epoxy resin, not observed in Figure 1e. All components were weighed prior to assembly, as detailed in Table 2.

### 3.4. Scanning Kelvin Probe (SKP)

The Scanning Kelvin Probe (SKP) is a noninvasive technique for determining the electrochemical surface analysis, particularly sensitive to conductive materials. Although conceptually related to atomic force microscopy (AFM), it operates without physical contact, probing the electric potential difference between a vibrating probe and the sample [64]. In this study, the SKP was employed to estimate the work function of Inconel 625, both prior to device assembly, to evaluate its appropriateness as a positive or negative CC, and in *post-mortem* analysis to investigate whether electrochemical alterations occurred after contact with the electrolyte and one year cycling performance. The underlying principle relies on the equalization of Fermi levels (electrochemical potentials) between the probe tip and the material surface, a phenomenon that is key for characterizing electrochemical equilibrium at interfaces and is particularly relevant in the context of energy storage devices, as proposed in this work.

The technique allows for mapping the contact potential difference (CPD) with micrometric resolution, demonstrating local variations in surface potential ϕ, which correlates to the work function (WF). To ensure a constant distance between the probe tip and the sample during acquisition, the Capacitive Tracking Measurement (CTM) method was employed. This approach dynamically adjusts the position of the probe in response to surface topography, ensuring accurate potential measurements throughout the scan [64]. In this study, the distance between the probe and the sample was fixed at 100 μm with the system’s positioning and operation continuously monitored using an integrated micro camera. The spatial resolutions were set to 50 µm/point along the x direction and 150 µm/point along the y direction. The insulated tungsten tip chemical potential μ is −4.44 eV in the absolute chemical potential scale where the zero is set for the electrons at rest in a vacuum. It is worth highlighting that the chemical potential of the standard hydrogen electrode (SHE) is μ(SHE) = −4.44 eV; therefore, μ(W) = 0 eV vs. SHE.

### 3.5. Electrochemical Performance Measurements

Potentiostatic Electrochemical Impedance Spectroscopy (PEIS) is a non-invasive technique in which a potentiostat applies a low-amplitude AC signal (10 mV) superimposed on a constant DC bias, allowing for the measurement of the system’s impedance response across a broad frequency spectrum. This approach offers valuable insights into electrochemical processes occurring at the interfaces between the cell components, particularly those related to charge transfer resistance and ionic conductivity. In this study, four impedance scans were conducted over a frequency range of 7 MHz to 100 mHz to ensure the reproducibility and consistency of the results. The impedance data were fitted to an equivalent circuit model using *EC-Lab^®^* V11.60 software to extract characteristic electrochemical parameters.

Cyclic voltammetry (CV) is a widely employed electrochemical technique used to investigate the redox behavior of materials within a cell. It involves the linear variation in the applied potential between two predefined limits at a constant scan rate, while recording the resulting current. In this study, the voltage was swept from –0.4 to 2 V at a scan rate of 10 mV.s^−1^. The analysis of the resulting voltammograms provides valuable insights into oxidation and reduction mechanisms, electrochemical kinetics, and the reactivity of the cell components under the selected conditions.

The charge/discharge analyses were designed with a 15 min charging step under constant voltage (CVoltage) conditions. The discharge phase was performed by connecting an external resistor in parallel with the device terminals. This configuration was chosen to enable precise control over the discharge process, independent of the potentiostat amplifier and unaffected by possible fluctuations in the cell’s internal resistance. Additionally, a continuous discharge test was implemented to investigate the periodic voltage oscillations observed during this step. This phenomenon was monitored to better understand the dynamic behavior of the cell during the discharge step.

PEIS and CV measurements were performed using a *Biologic VMP-300* (Seyssinet-Pariset, France) potentiostat/galvanostat/impedance spectroscope, while the charge/discharge tests were conducted on a *Neware CT-4008Tn 5 V/20 mA* battery tester (Kowloon Bay, HongKong, China).

The data collected in the laboratory was transferred to *OriginPro 2025^®^* (Learning Edition), OriginLab Corporation, Northampton, MA, USA.

### 3.6. SEM/EDX Analysis and Samples Preparation

Scanning Electron Microscopy coupled with Energy-Dispersive X-ray Spectroscopy (SEM/EDX) was employed to characterize the surfaces of both the positive (Inconel 625) and negative (Aluminum) CC. SEM/EDX is a well-established technique for surface morphology imaging and elemental analysis, based on the interaction between a focused electron beam (operated at 15 keV) and the sample’s atoms. When the beam strikes the sample, it induces atomic excitation, leading to the emission of characteristic X-rays that can be detected and analyzed to determine the qualitative and quantitative elemental composition. This technique enables the identification of most elements in the periodic table, except for hydrogen (H), helium (He), and lithium (Li). The analyses were carried out using a high-resolution environmental SEM equipped with X-ray microanalysis and electron backscattered diffraction capabilities: the FEI Quanta 400 FEG ESEM/EDAX Genesis X4M system.

After undergoing various electrochemical analyses, the device was disassembled to evaluate the condition of the CC following contact with the solid electrolyte. The Inconel 625 component was carefully cleaned using only compressed air, to remove residual electrolyte powder without introducing contaminants. The aluminum container was sectioned to enable analysis of both its inner and outer surfaces. No conductive coating was required for either sample, as both materials were inherently conductive.

### 3.7. X-Ray Diffraction (XRD) Analyses

X-ray diffraction analyses of the Aluminum CC (interior and exterior) metallic cut samples were performed on a Bruxer D6 Phaser with Bragg–Brentano geometry with CuKα radiation (1.54 Å). Scan type 2θ from 5 to 70° and 0.017° step. The diffractogram was analyzed using Match! 3.11.2.188 64-bit.

### 3.8. First Principles Simulations for INCONEL 625

Density functional theory (DFT) as implemented in VASP [69,70] and GGA-PBE exchange correlation was used to optimize the bulk crystalline and surfaces of Inconel with different stoichiometries and surface orientations. The final composition of the Inconel 625 was Ni_41_Cr_15_Mo_3_Fe_2_AlSiNb, which totals 64 atoms. The composition was based on the SEM/EDX analyses and the correspondent number of atoms of each element was chosen to match the composition but so that the final structure could still be manageable in terms of calculation time. The structure was optimized from the Ni cubic face centered structure. Inconel 625 is a nickel-based superalloy whose matrix is essentially a face-centered cubic (FCC) Fm-3m (sg. 225) γ-Ni solid solution. The composition of Inconel 625 is ~58 wt% Ni, ~20–23 wt% Cr, ~8–10 wt% Mo, ~3.5 wt% Nb + Ta, small amounts of Fe, Co, etc. Herein the composition is 64.4 wt% Ni, 20.9 wt% Cr, 7.7 wt% Mo, 3.0 wt% Fe, 2.5 wt% Nb, 0.75 wt% Si, and 0.72 wt% Al and the elements randomly substitute the Ni in the pure FCC structure. The simulated Inconel is a half-metallic ferromagnet with total magnetic moment of 11.9486 Bohr magnetons (µ_B_). Its density is 8.474 g.cm^−3^ and the optimized supercell (P1) have a crystal structure with lattice parameters a= 7.106 Å, b = 14.390 Å, c = 7.154 Å, and α = 90.061°, β = 89.843°, and γ = 89.946°.

Due to magnetic moments in the model, a spin-polarized magnetic calculation using ‘accurate’ precision and a default planewave cutoff energy of 293.235 eV used as the Ni pure structure had been optimized previously. The electronic iterations convergence is 1.00 × 10^−5^ eV using the Normal (blocked Davidson) algorithm and reciprocal space projection operators. The requested k-spacing is 0.5 Å^−1^, which leads to a 2 × 1 × 2 mesh. This corresponds to actual k-spacings of 0.446 × 0.446 × 0.446 Å^−1^. The k-mesh is forced to be centered on the gamma point. Using first order Methfessel-Paxton smearing with a width of 0.2 eV. For electrical and surface simulations, we had to impose the non-magnetic model.

Other compositions such as Ni_19_Fe_2_Cr_7_Mo_3_Nb (~56 wt% Ni, ~18 wt% Cr, ~15 wt% Mo, ~6 wt% Fe, ~5 wt% Nb) were also optimized and the total local potential was simulated to obtain the work function, which resulted very similar to the obtained herein. We have also performed ab initio molecular dynamics to obtain the simulated annealing structure from the liquidus; however, the result was an amorphous structure, which was not intended and therefore not pursued.

Overall, after the optimization of the crystal structure, the total chemical potential was simulated after obtaining the (100), (010), (001) and (101) surfaces, as the Inconel is a solid solution, and, therefore, there is not one solution but different possibilities for the structure and surfaces.

The electrical properties [71] including electronic band structure, Fermi surface, and electrical and thermal conductivities were obtained with the bulk structure.

## 4. Conclusions

This study provides a comprehensive clarification of the mechanisms responsible for battery-like behavior in all-solid-state devices fabricated without conventional Faradaic electrodes. By decoupling ionic transport from electrode redox chemistry, we demonstrate that the origin of the observed discharge plateau and long-term energy delivery lies in interfacial electrostatics rather than bulk electrode reactions. Specifically, the difference in electron chemical potential between the two CCs governs the spontaneous discharge process, while the solid electrolyte—behaving as an electronic insulator—forces charge compensation to occur through the formation of electrostatic double layers at the interfaces. The persistent plateau voltage emerges from electron tunneling and electric-field-driven reduction of cations previously accumulated at the electrolyte surface, confirming that energy can be stored and released without participation of traditional intercalation or conversion electrodes. These results establish the concept of a “capacity-less” battery, in which at least one interface, rather than an active electrode, serves as the primary reservoir of electrochemical energy.

In parallel, this work highlights the value of integrating additive manufacturing into the design of next-generation solid-state energy storage and harvesting devices. Inconel 625, processed through metal Fused Filament Fabrication (FFF), proved to be a highly stable and compatible CC when paired with the ferroelectric electrolyte Na_2.99_Ba_0.005_OCl and aluminum as the counter electrode. The design flexibility inherent to FFF enabled the fabrication of optimized geometries that increased the effective contact area between components and improved electrostatic coupling across the device. Electrochemical characterization using PEIS, CV, and charge/discharge cycling confirmed the stability of the system and revealed dynamic behavior consistent with interface-dominated energy storage.

Long-term testing under ambient conditions demonstrated the excellent chemical and mechanical integrity of the Inconel 625 CC, which exhibited no detectable degradation after one year of continuous operation. In contrast, the aluminum electrode, while chemically stable, displayed mechanical wear on the surface in contact with the electrolyte, likely associated with minor moisture retention in the solid electrolyte stack. These results underscore the superior durability of Inconel 625 and its suitability for devices designed to operate in demanding environments.

The performance, stability, and corrosion resistance of Inconel 625 open opportunities for large-area or in situ fabricated solid-state devices in extreme or specialized settings. Potential applications include marine and subsea sensing platforms, molten-salt or Na–S batteries operating near 1000 °C, geothermal power devices exposed to brines or steam, aerospace systems requiring thermal-shock-resistant components, nuclear or industrial infrastructures needing radiation-tolerant power units, and desert-field installations where extreme thermal cycling limits the use of conventional metals. The ability to produce custom geometries directly via additive manufacturing further strengthens the adaptability of this alloy to structural, embedded, or multifunctional energy-storage applications.

Overall, the present work reinforces two major conclusions. First, interfacial electrostatics—rather than redox chemistry—can fully sustain the energy storage behavior of certain all-solid-state systems, establishing a new conceptual framework for “capacity-less” solid-state batteries. Second, Inconel 625 fabricated through metal FFF emerges as a practical, stable, and design-flexible CC for solid electrolytes, offering new avenues for the development of durable, interface-engineered solid-state energy harvesters and batteries. Together, these findings lay a foundation for the rational design of robust, multifunctional electrochemical devices capable of operating in harsh environments and contributing to the next generation of sustainable energy technologies.

## Figures and Tables

**Table 2:** Precision mass of each component used in the assembly of the all-solid-state battery, determined inside an argon filled glovebox.

Element:	*Al Container*	*Inconel + Cu Tab*	*Na_2.99_Ba_0.005_OCl*	*Total Device*
Mass (g):	46.871	19.454	27.878	94.203

## Data Availability

Data is available on reasonable request.

## Appendix A

**Table A1:** Electrochemical performance as a function of external resistor, including the average discharge potential difference and current, as well as the specific capacity per cycle. The discharges were obtained after a 15 min constant voltage charge, except in the case of 6.8 MΩ resistor in which discharge was initiated without previous external charge.

**Summary of the 64 Discharge Cycles of the Device (6477 h in Total)** **After a 15 min Constant Voltage Charge (Except for 6.8 MΩ Resistor)** **A = 15 cm^2^**					
**External Resistors (kΩ)**	**Cycle**	**Discharge Time (h)**	**Average Discharge Potential (V)**	**Average Discharge Current (mA)**	**Discharge Capacity (mA.h)**
6800	1st	532	1.00	0.0001	0.078
1000	2nd	1505	0.98	0.001	1.484
555	3rd	48	1.03	0.002	0.089
	4th	48	1.03	0.002	0.089
	5th	48	1.03	0.002	0.089
	6th	48	1.04	0.002	0.090
	7th	48	1.04	0.002	0.090
46.4	8th	48	1.02	0.022	1.057
	9th	48	1.02	0.022	1.051
	10th	48	1.02	0.022	1.058
	11th	48	1.03	0.022	1.066
	12th	48	1.04	0.022	1.072
21.7	13th	48	1.03	0.048	2.287
	14th	48	1.03	0.047	2.277
	15th	48	1.02	0.047	2.267
	16th	48	1.02	0.047	2.256
	17th	48	1.02	0.047	2.253
5.64	18th	48	0.47	0.084	4.019
	19th	48	0.48	0.085	4.064
	20th	48	0.47	0.084	4.038
	21st	48	0.48	0.084	4.051
	22nd	48	0.48	0.085	4.065
	23rd	48	0.47	0.084	4.013
	24th	48	0.46	0.082	3.956
	25th	48	0.51	0.090	4.317
	26th	48	0.46	0.082	3.918
	27th	48	0.49	0.086	4.149
99.4	28th	48	0.86	0.009	0.417
	29th	48	0.90	0.009	0.435
	30th	48	1.04	0.010	0.501
	31st	48	1.04	0.011	0.504
	32nd	48	1.05	0.011	0.507
	33rd	48	1.05	0.011	0.508
	34th	48	1.05	0.011	0.509
	35th	48	1.05	0.011	0.509
47.0	36th	749	1.05	0.022	16.641
	37th	48	1.04	0.022	1.063
	38th	48	1.05	0.022	1.068
	39th	48	1.05	0.022	1.073
	40th	48	1.05	0.022	1.075
	41st	48	1.05	0.022	1.077
	42nd	48	1.05	0.022	1.076
	43rd	48	1.05	0.022	1.071
	44th	48	1.05	0.022	1.075
21.7	45th	48	1.03	0.048	2.287
	46th	48	1.04	0.048	2.300
	47th	48	1.03	0.048	2.291
	48th	48	1.04	0.048	2.290
9.74	49th	48	0.96	0.099	4.738
	50th	48	0.90	0.092	4.411
	51st	48	0.86	0.088	4.224
	52nd	48	0.88	0.090	4.331
	53rd	48	0.89	0.091	4.386
	54th	48	0.85	0.088	4.211
	55th	48	0.86	0.089	4.250
	56th	48	0.86	0.088	4.226
	57th	48	0.86	0.088	4.237
	58th	48	0.84	0.086	4.134
	59th	48	0.87	0.089	4.270
	60th	48	0.86	0.088	4.239
	61st	48	0.84	0.087	4.164
	62nd	48	0.85	0.087	4.179
	63rd	500	0.84	0.087	43.342
	64th	359	0.91	0.094	33.546
